# Do Foliar, Litter, and Root Nitrogen and Phosphorus Concentrations Reflect Nutrient Limitation in a Lowland Tropical Wet Forest?

**DOI:** 10.1371/journal.pone.0123796

**Published:** 2015-04-22

**Authors:** Silvia Alvarez-Clare, Michelle C. Mack

**Affiliations:** Department of Biology, School of Natural Resources and the Environment, University of Florida, Gainesville, Florida, United States of America; The University of Auckland, NEW ZEALAND

## Abstract

Understanding nutrient limitation of net primary productivity (NPP) is critical to predict how plant communities will respond to environmental change. Foliar nutrients, especially nitrogen and phosphorus concentrations ([N] and [P]) and their ratio, have been used widely as indicators of plant nutritional status and have been linked directly to nutrient limitation of NPP. In tropical systems, however, a high number of confounding factors can limit the ability to predict nutrient limitation —as defined mechanistically by NPP responses to fertilization— based on the stoichiometric signal of the plant community. We used a long-term full factorial N and P fertilization experiment in a lowland tropical wet forest in Costa Rica to explore how tissue (foliar, litter and root) [N] and [P] changed with fertilization, how different tree size classes and taxa influenced the community response, and how tissue nutrients related to NPP. Consistent with NPP responses to fertilization, there were no changes in community-wide foliar [N] and [P], two years after fertilization. Nevertheless, litterfall [N] increased with N additions and root [P] increased with P additions. The most common tree species (*Pentaclethra macroloba*) had 9 % higher mean foliar [N] with NP additions and the most common palm species (*Socratea exohrriza*) had 15% and 19% higher mean foliar [P] with P and NP additions, respectively. Moreover, N:P ratios were not indicative of NPP responses to fertilization, either at the community or at the taxa level. Our study suggests that in these diverse tropical forests, tissue [N] and [P] are driven by the interaction of multiple factors and are not always indicative of the nutritional status of the plant community.

## Introduction

Understanding nutrient limitation of net primary productivity (NPP) is critical to predict how plant communities will respond to environmental changes, such as nitrogen deposition and higher carbon dioxide atmospheric levels [1,2,3]. Foliar nutrients, especially nitrogen and phosphorus concentrations ([N] and [P]) and their ratio, have been used widely as indicators of plant nutritional status and have been linked directly to nutrient limitation of NPP [4,5,6,7,8]. Nutrient concentrations in leaf litter, and to a lesser extent of fine roots, have also been used to predict global patterns of soil nutrient availability and biogeochemical cycling [9,10,11,12]. Absolute and relative plant [N] and [P] have been shown to predict soil nutrient availability most accurately in certain instances. For example, N:P ratios are good predictors of soil nutrients at large spatial scales, where large variation in soil and plant parameters across biomes overpowers local variance (for example see [9,13,14]), and in low diversity systems with relatively constrained variability in N:P ratios [15,16]. In tropical systems, however, using plant [N] and [P] to assess NPP can be challenging [8,17,18,19]. In diverse tropical forests, [N] and [P] can vary widely within small spatial and temporal scales and can reflect factors different from soil nutrient availability. For example, seasonality [17], life history traits [20], species identity [21], and herbivory [22] can also influence [N] and [P], thereby decoupling the community N:P signal from relative nutrient availability. The decoupling of the N:P signal makes interpreting foliar (and other tissue) N:P ratios challenging because of partitioned control between environmental and physiological factors [7].

Nutrient limitation is mechanistically defined as an increase in growth (or biomass accumulation) with increased nutrient availability [23,24]. Thus, to effectively elucidate the relationship between soil nutrient availability and nutrient concentrations in plant tissues in a given system, values can be calibrated by comparing them to NPP responses to fertilization [18,23,25]. This has been rarely done in tropical systems, partly because few fertilization experiments exist in the tropics. Results from the few existing studies suggest that foliar P is more responsive to nutrient additions than N, and that responses are site and species specific [16,20,25,26,27]. Because to our knowledge only one fertilization experiment has been conducted in a lowland tropical wet forest [28], we used a full factorial NP fertilization experiment conducted in a lowland tropical wet forest in Costa Rica [29] to test if [N] and [P], and their ratio in leaves, litter and roots were good indicators of nutrient limitation. In this forest, high soil nutrient levels and limited NPP responses to N and P fertilization [29] suggest that biological factors may play a dominant role in determining fertilization responses. Thus, we focused on species identity and tree-size class to address the following questions:

### (1) How does N and P fertilization affect foliar, litter, and root nutrients?

Because we did not observe a strong NPP response to fertilization after 2 years [29], we predicted that fertilization would result in “luxury consumption”, or increased nutrient concentrations in all measured tissues by the second-year collection [30]. High [N] and [P] in green leaves are typically associated with higher concentrations in litter and roots [10,21,31,32,33] and thus, we predicted [N] and [P] increases in these tissues as well. Because our scale was at the “community level” and we could not assure that leaves and roots were coming from the same functional groups of plants (e.g. root mats from understory palms versus leaves from trees), we did not compare among tissues.

### (2) How does tree size and taxa influence foliar nutrient concentrations and their response to fertilization?

After 2 years of fertilization, we observed a strong growth response to P additions for small (5–10 cm diameter at breast height (DBH)) trees but not for large (>10 cm DBH) trees [29]. This proved that small trees were effectively accessing the added fertilizer; therefore we predicted a consistent increase in foliar [P] in small trees by the second year foliage collection. From selected taxa, we only observed an increase in growth after P and NP additions in the most abundant canopy palm (*Socratea exohrriza*). Therefore, we expected a consistent increase in foliar P for this palm.

### (3) Is there a direct relationship between soil or plant nutrients and NPP within our study site?

We did not find a strong community-level NPP response with either N or P additions after 2 years [29]. Thus, if indeed plant tissue nutrient concentrations and their ratios reflect soil nutrient availability in this forest, we expected to find mean foliar N:P ratios between 14 and16, which according to stoichiometric theory indicate that neither N or P strongly limit NPP in this forest [34,35].

## Materials and Methods

### Site description

The study was conducted at a private forest reserve within EARTH University (www.earth.ac.cr), located in Guácimo, Limón, Costa Rica (10° 11’ N and 84° 40’ W). Permission to conduct research at the site was granted by EARTH University’s Office of Investigations and by the Ministerio de Ambiente y Energía (MINAE) in Costa Rica, under research permit # 01872 to SAC. No human or animal subjects were used for this experiment. No endangered species of plants were used either. No other permits were required. This site is located approximately 30 m above sea level and consists of 900 ha of mature and regenerating rainforest and wetlands. Mean annual temperature is 25.1°C and mean annual precipitation (MAP) is 3,464 mm. Soils in the area are from volcanic origin, high in clay, with poor drainage, and have been classified as inceptisols and ultisols [36]. Total N and P, and macronutrient concentrations are on the higher end of the spectrum, relative to other lowland tropical forests (Table 1). In addition, tree growth, litter productivity, and root growth index in this forest are high, contributing to the notion that–for a lowland tropical forest—this is a relatively fertile system (Table 1).

**Table 1:** Mean (± SE) soil chemical parameters and indicators of net primary productivity (NPP) measured for each of the four fertilization treatments.

	Fertilization treatment			
	Control	+N	+P	+NP
**Soil parameters (0-10cm depth)**				
pH	4.32 ± 0.06	4.25 ± 0.05	4.13 ± 0.08	4.10 ± 0.08
Extractable P(μg g^-1^)^a^	3.10 ± 1.85	1.97 ± 0.75	3.42 ± 1.30	3.07 ± 0.66
Total P (μg g^-1^)	1690.00 ± 310.23	1495.28 ± 164.28	1646.00 ± 244.72	1661.67 ± 173.98
DIN (μg g^-1^)^b^	13.57 ± 0.73	13.87 ± 0.67	13.80 ± 0.70	13.95 ± 0.95
Total N (μg g^-1^)	438.33 ± 10.75	436.45 ± 4.81	445.50 ± 8.61	433.17 ± 10.52
Net N mineralization (μg N g^-1^ d^-1^)	1.50 ± 0.52	1.95 ± 0.40	1.22 ± 0.52	2.14 ± 0.34
Net nitrification (μg N g^-1^ d^-1^)	1.24 ± 0.34	1.37 ± 0.18	1.04 ± 0.32	1.21 ± 0.25
**Indicators of NPP**				
Total DBH increase (mm)^c^	260.39 ± 31.69	258.92 ± 10.17	265.97 ± 29.57	249.48 ± 68.02
Trees that grew^d^	66.32 ± 4.62	66.59 ± 1.94	76.80 ± 3.69	76.95 ± 3.36
RGR (mm mm^-1^ yr^-1^)^e^	0.018 ± 0.002	0.021 ± 0.002	0.023 ± 0.003	0.025 ± 0.006
Litterfall productivity (Mg C ha^-1^ yr^-1^)^f^	4.61 ± 0.51	4.72 ± 0.32	5.28 ± 0.77	4.43 ± 0.70
Fine root biomass (Mg C ha^-1^) ^g^	2.07 ± 0.42	1.70 ± 0.23	1.96 ± 0.25	2.35 ± 0.42
Root growth index (Mg C ha^-1^ yr^-1^)^h^	0.40 ± 0.10	0.32 ± 0.03	0.30 ± 0.09	0.40 ± 0.08

Samples were collected two years (for soil parameters) or 2.7 years (for NPP parameters) after initial fertilization. None of the treatment means were significantly different from the control (Dunnett’s Test, with significance of *P* < 0.05). For methodological details refer to [29].

^a^ Extracted with a Mehlich I solution [62].

^b^ Average dissolved inorganic nitrogen (DIN) calculated from the sum of nitrate and ammonium concentrations.

^c^ Average of total diameter at breast height (DBH) increase in each plot, calculated as Σ(DBH at 2.7 years)–Σ(DBH at the onset of the study) for all trees larger than 10 cm DBH and 10 small trees (5 cm >DBH> 10 cm) per plot.

^d^ Average of percent of trees in each plot that grew between 2–2.7 years after initial fertilization for the same trees as above.

^e^ Average relative growth rate (RGR) throughout the course of the study, calculated as the slope of a line fitted through the log-transformed diameter values at each tree census, for the same trees as above.

^f^ Average rate of foliar litterfall collected in mesh traps between 1–2 years after initial fertilization.

^g^ Average root biomass for 0-10cm deep cores collected in each plot, 2 years after initial fertilization.

^h^ Average fine root growth calculated from ingrowth cores, which were installed 10 cm deep, at the onset of the study and were removed 2 years after initial fertilization.

Soils, climate, floristic composition and tree density at the EARTH Forest Reserve are similar to those from the alluvial soils at La Selva Biological Station, a well-studied forest in Costa Rica [37]. Both forests are dominated by the legume tree *Pentaclethra macroloba*, which contributed almost 30% of the total basal area within our study plots. High palm density is also characteristic of forests in the Caribbean lowlands of Costa Rica [38,39]. At EARTH Forest, the second most important species is *Socratea exorrhiza*, a single-stem canopy or subcanopy palm with large leaves and distinctive stilt roots. In contrast with *Pentaclethra*, however, this palm is important because of the high frequency and density in which it occurs at EARTH, and not because of a large basal area.

### Experimental design

In May 2007, we established 24–30 x 30 m plots within mature, non-flooding areas of the reserve and assigned them randomly, in a complete block design (n = 6), to three fertilizer treatments or a control. All plots were separated from each other by at least 100 m. Since the onset of the experiment, fertilizer was broadcast by hand twice a year on the surface of the 900 m^2^ plots to implement the following treatments: +P (47 kg ha^-1^yr^-1^ of P as super triple phosphate), +N (100 kg ha^-1^yr^-1^ of N applied as ammonium nitrate and urea), and +NP (N and P added together in quantities as in +N and +P plots). All measurements were restricted to the central 400 m^2^ of each plot (20 x 20 m) to reduce edge effects. At the onset of the experiment and 1 and 2 years after fertilization, we measured several physical and chemical soil parameters (Table 1). Methodological details for these analyses can be found in [40]. Net primary productivity (NPP) was measured continuously for 2.7 years after initial fertilization. All trees larger than 10-cm DBH and ten trees between 5 and 10 cm DBH were marked and identified in each plot. Tree diameter increase measurements were conducted every six months using band dendrometers and DBH tapes [41]. Litterfall productivity was measured using two litterfall traps per plot, which were emptied every two weeks. Root biomass and growth were measured from soil samples and with ingrowth cores, which were installed 10cm deep, at the onset of the experiment and were removed after two years of fertilization (Table 1; [29,41,42]). Although plot-averaged NPP and soil parameters were not different from the control after 2 years (Table 1), there was a significant increase in soil extractable P, Total P, and proportion of tree growth with P additions when comparing values for the same plot before fertilization and two years after fertilization (T2-T0) [29].

### Foliage, litterfall and root collection

Foliage, litterfall and root samples were collected from each plot between July 2007 and September 2009 prior to fertilization, and 1 and 2 years after initial fertilization. To standardize the foliage collected, in each plot we collected fully expanded, sun-leaves from common tree species. We tried to collect samples from the same species in each plot but because of the high diversity characteristic of this forest, this was not always possible (please refer to S1 Table to see the percent of plots in which each of the “common species” were found). We used a pole pruner, or crossbow with a bolt affixed with monofilament line. One sample was composed of a group of at least ten leaves per tree placed in a bag. Overall, each year we collected foliar samples from 286 trees representing 36 genera and 46 species. When possible, samples were collected from the same 4–8 large trees (>10 cm DBH) and 4–8 small trees (4–9 cm DBH) per plot. When it was not possible to collect leaves from the same trees (because a tree had died, lost its leaves or showed substantial herbivory), foliage was collected from another tree in the same plot and size category. To obtain a mean community value, we averaged all trees for each collection time. To evaluate the effects of fertilization at a finer scale, we selected six common taxa representing several functional groups. However, because of the high diversity and low frequency of conspecific trees in this forest, in some cases we grouped several congeners and categorized them as “taxa” for statistical analysis at this scale (S1 Table).

Litterfall was collected biweekly through the duration of the study using two 0.25 m^2^ mesh traps per plot supported by a polyvinyl chloride (PVC) frame as described in [42]. Three representative subsamples from the litterfall collected during the second year were chemically analyzed. The first subsample (pre-fertilization) included foliar litterfall collected between 23 August and 9 September 2007. The second subsample (one year after fertilization) included foliar litterfall collected between 8 October and 5 November 2008, and the third subsample (two years after fertilization) included foliar litterfall collected between 9 July and 10 August 2009. Each of these subsamples was separated into species, when possible, and the remaining leaves analyzed collectively. Finally, we used a pound core inserted 15 cm into the ground to collect two root samples per plot, at pre-selected random locations where no other soil samples had been taken. We then combined these cores to obtain a composite sample per plot. Samples were collected prior to fertilization, and one and two years after initial fertilization, and root biomass determined by washing samples with a sieve, followed by drying and weighing, as described in [29].

### Nutrient analysis

We prepared foliar, litterfall, and roots for chemical analyses by drying samples at 60°C and grinding them to a fine powder. Total percent N and C was measured with an elemental analyzer (ECS 4010, Costech Analytical, Valencia, CA, USA). Total P was measured using ash digestion [43] followed by colorimetric determination of ortho-phosphate on a microplate reader (PowerWave XS Microplate Reader, Bio-Tek Instruments Inc., Winooski, VA, USA). To test for changes in leaf physical properties resulting from fertilization, and to analyze nutrient concentrations on an area basis, we scanned five fresh leaves per sample, measured their area, then dried and weighed the leaves, and calculated specific leaf area (SLA) (cm^2^ g^-1^). This was done for foliar samples collected one and two years after initial fertilization.

### Statistical analysis

To evaluate the effect of fertilization on community-level foliar, litter and root chemistry over the course of the experiment, and of foliar chemistry of trees from different sizes and taxa, we used plot-averaged values (n = 24) in repeated measures MANOVAs. We used time-specific measurements as stepwise dependent variables, and treatment and block as independent variables. When there was a significant time*treatment interaction, we conducted Dunnett’s ANOVAs for the second sampling period (two years after fertilization) to test if concentrations had changed after a biologically-significant period. We selected this approach over univariate repeated measures ANOVA because in some cases the assumption of sphericity was not met [44]. For root [P], we excluded data from plot 19 because it was an extreme outlier (Cook’s D distance > 4/n; [45]).

To test the effects of fertilization on litter chemistry from different taxa, we analyzed separately *Pentaclethra macroloba* (the most common species) and a species group (termed “Tiliaceae”), which included primarily *Goethalsia meiantha* with traces of *Apeiba membranaceae* or *Luehea seemannii* (which could not be visually separated from *Goethalsia*). We analyzed the relationship between indicators of NPP and soil or plant chemistry measurements with non-parametric Spearman correlation coefficients. All analyses were conducted in JMP 8.0 (SAS Institute Inc., Cary, NC, USA).

## Results

### Effects of fertilization on foliar, litter, and root N and P concentrations

**Fig 1:**
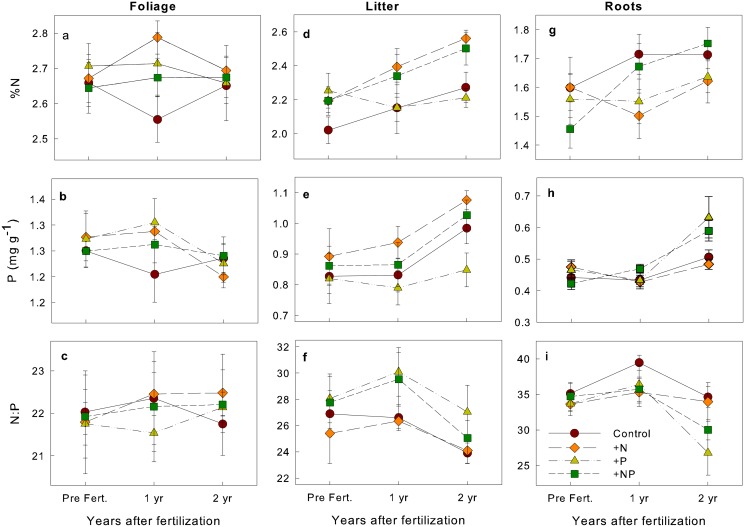
Mean (± SE) nutrient concentrations and N:P ratios over time for the four fertilization treatments in (A-C) leaves, (D-F) litter, and (G-I) roots. Results from repeated measures MANOVAs are shown in S2 Table.

**Fig 2:**
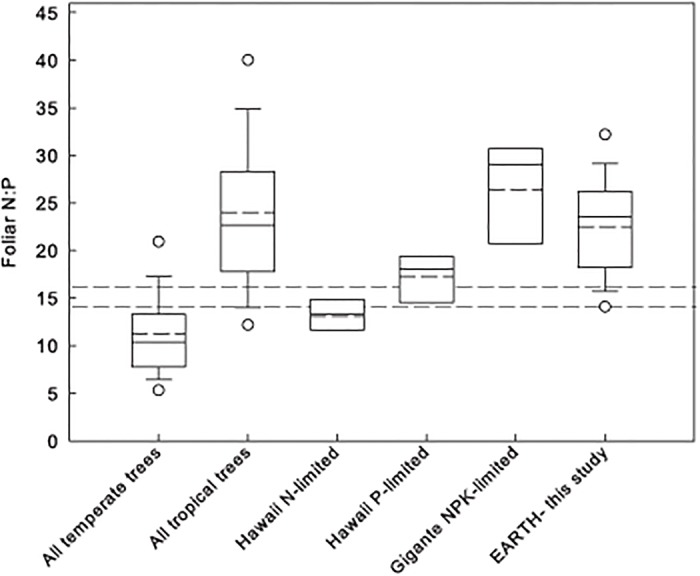
Box plots showing foliar N:P ratios at EARTH Forest relative to other forests. The dashed line is the mean, the 25^th^ and 75^th^ percentiles are encased in the box, the whiskers are the 10^th^ and 90^th^ percentiles and the dots are the 5^th^ and 95^th^ percentiles. Data for all temperate and tropical trees are from [17,20]. Hawaii data are from [16]. For the last four sites, each data point was a species mean across control plots. Dashed lines indicate the 14–16 N versus P limitation threshold.

At the community level, there were no differences among treatments or sampling times in foliar [N] and [P] or N:P ratios (Fig 1; S2 Table). In addition, when comparing the change in foliar nutrients two years after initial fertilization (two year minus pre fertilization values), there was no significant difference among treatments in foliar N (treatment *F*_3,23_ = 0.38, *P* = 0.77; block *F*_5,23_ = 0.76, *P* = 0.60) or P (treatment *F*_3,23_ = 0.89, *P* = 0.47; block *F*_5,23_ = 0.72, *P* = 0.63). Specific leaf area also did not differ among treatments (treatment *F*_3,21_ = 1.10, *P* = 0.38; block *F*_5,21_ = 0.43, *P* = 0.82). Foliar N:P ratios did not change after two years of fertilizations and were remarkably variable, with values overlapping N-limited, P-limited and NPK-limited forests (Fig 2).

Overall, litterfall nutrient concentrations were high but varied markedly with time (Fig 1D–1F; S2 Table). After two years, the N and NP treatments had higher mean litterfall [N] than the control or P plots (Fig 1D; S2 Table). There were no differences among treatments for mean litterfall [P] or litterfall N:P ratios (Fig 1E and 1F; S2 Table). Finally, there was no significant difference among treatments in root [N] but roots in the P and NP treatments had higher [P] two years after fertilization, as revealed by a significant time*treatment interaction (Fig 1H; S2 Table).

### Influence of tree size class and taxa on responses to fertilization

**Fig 3:**
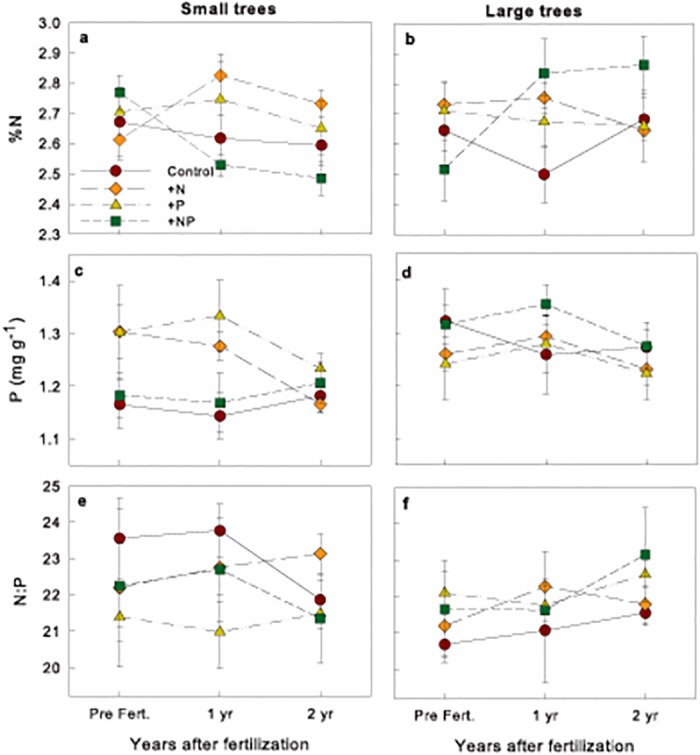
Mean (± SE) nutrient concentrations and N:P ratios over time for the four fertilization treatments for (A, C, E) small (5–10 cm DBH) and (B, D, F) large (>10 cm DBH) trees. Results from repeated measures MANOVA are shown in S3 Table.

Overall, large trees (>10 cm DBH) had higher foliar [P] (*t* = 2.28, *P* = 0.02) but not [N] (*t* = 0.082, *P* = 0.41) than small trees (5–10 cm DBH), both before and two years after fertilization. In addition, two years after fertilization, large trees had 48.7% higher N and 45.0% higher P per unit leaf area than small trees across treatments (N: *t* = 6.89, *P* <0.01, P: *t* = 6.72, *P* <0.01). As revealed by a significant time*treatment interaction (S3 Table), two years after initial fertilization, large trees had higher mean foliar N in the +NP treatment, although this result was not consistent across blocks (Fig 3B; S3 Table). Two years after initial fertilization there was no difference in mean foliar P in small or large trees (Fig 3C and 3D; S3 Table). Foliar N:P ratios did not vary among treatments or between size classes through the course of the experiment (Fig 3E; S3 Table).

**Fig 4:**
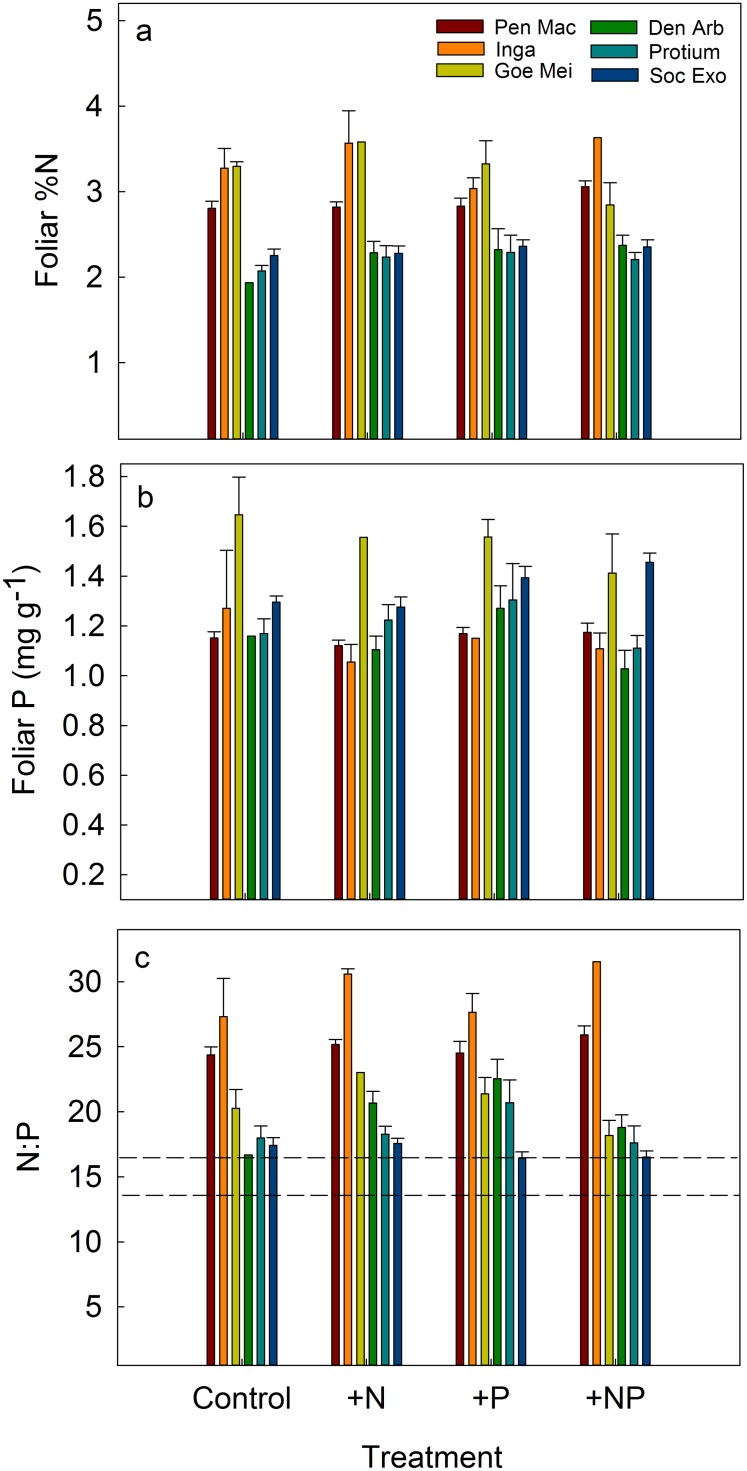
Mean (± SE) foliar N and P concentrations, and N:P ratios in the four fertilization treatments for six common taxa, including two legumes (*Inga* and *Pen Mac*) and a canopy palm (*Soc Exo*). Asterisks indicate differences from the control (Dunnett’s test; * = *P* < 0.1, ** = *P* < 0.05). For full species names and life-history traits refer to S1 Table.

**Fig 5:**
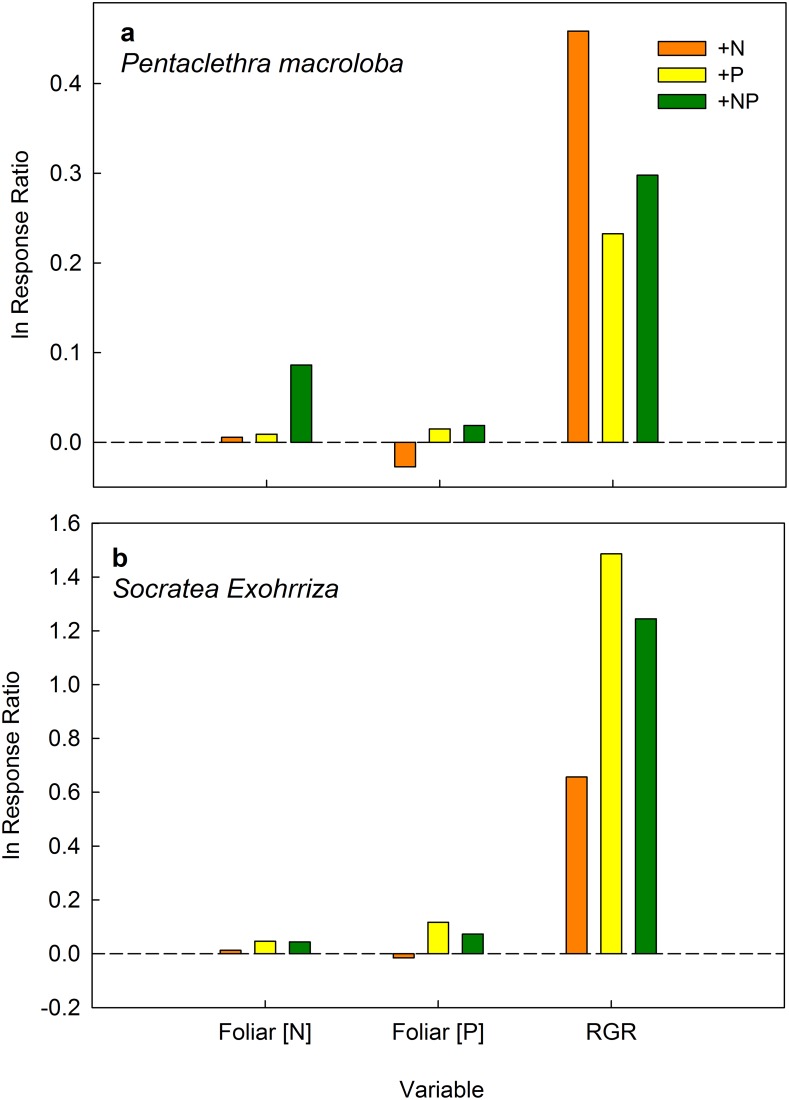
Changes in foliar nutrient concentrations and relative growth rate (RGR) for the fertilization treatments relative to the unfertilized controls for (A) *Pentaclethra macroloba* and (B) *Socratea exorrhiza*. Notice Y axis difference between panels.

We compared foliar and litter nutrients among four species and two genera where replicates were sufficient to conduct statistical analyses (S1 Table). Two years after initial fertilization, foliar N, P and N:P ratios differed significantly among taxa (S4 Table) but only two taxa differed among treatments in foliar N or P (Fig 4). Trees from *Pentaclethra* had 9% higher mean foliar N in the +NP treatment (Fig 4A), and *Socratea* palms had 15% and 19% higher mean foliar P relative to the control in the +P and +NP treatments, respectively (Fig 4B). There were no treatment effects in litter nutrients from *Pentaclethra* (*F*_3,13_ = 1.27, *P* = 0.34) or the Tiliaceae group (*F*_3,23_ = 1.87, *P* = 0.25). In sum, there were significant but contrasting responses between the two most abundant species of trees at EARTH forest: *Pentaclethra* responded mainly to N additions by increasing relative growth rate (RGR) and foliar N (in +NP plots), and *Socratea* responded mainly to P additions by increasing RGR and foliar P (Fig 5). Foliar N:P ratios differed among treatments for *Dendropanax* but this result was based on one individual and should therefore be interpreted with caution (Fig 4C). For the rest of the study species N:P ratios did not differ among treatments, blocks or sampling times.

### Relationship between soil or plant nutrients and NPP

We explored the relationship between plot-averaged soil- or plant-tissue chemistry and indicators of NPP across treatments. Foliar [N] and [P] were positively correlated with total DBH increase, but not with other indicators of stem growth (Table 2). Interestingly, foliar [P] was negatively correlated with litterfall productivity. From the litter chemistry parameters, only litter P was correlated with RGR. Root chemistry parameters were the best correlates of fine root biomass and growth. Root [P] was positively correlated with proportion of tree growth but negatively correlated with fine root biomass and productivity. Soil P—both Mehlich-extractable and total P—were also negatively correlated with root growth index.

**Table 2:** Spearman correlation coefficients showing the relationship between components of NPP and soil, foliar, litter and root chemistry.

	Indicators of NPP					
	Total DBH increase (mm)	Trees that grew	RGR(mm mm^-1^ yr^-1^)	Litterfall productivity (Mg C ha^-1^yr^-1^)	Fine root biomass (Mg C ha^-1^)	Root growth index (Mg C ha^-1^ yr^-1^)
**Soil parameters**						
pH	0.19	-0.34	-0.03	-0.16	-0.20	-0.23
Extractable P (μg g^-1^)	0.21	0.24	0.38*	-0.24	-0.23	-0.41*
Total P (μg g^-1^)	0.02	-0.04	0.05	-0.27	-0.18	-0.47**
DIN (μg g^-1^	-0.43*	-0.08	-0.28	-0.01	0.23	0.25
Net nitrification (μg g^-1^ d^-1^)	-0.31	-0.42*	-0.23	-0.44*	0.02	-0.06
**Foliar chemistry**						
Foliar N (%)	0.53**	-0.10	0.31	-0.11	-0.34	-0.30
Foliar P (mg g^-1^)	0.48*	0.24	0.32	-0.42*	-0.11	-0.25
Foliar N:P	-0.13	-0.08	0.01	0.43*	-0.01	-0.13
**Litter chemistry**						
Litter N (%)	0.27	-0.34	0.12	-0.04	-0.35	-0.01
Litter P (mg g^-1^)	0.26	-0.33	0.38*	0.05	-0.35	-0.24
Litter N:P		0.14	-0.31	0.12	0.23	0.48*
**Root chemistry**						
Root N (%)	0.11	0.24	-0.20	0.39*	0.36	0.17
Root P (mg g^-1^)	0.31	0.49**	0.35	-0.19	-0.42*	-0.38*
Root N:P	-0.25	-0.40*	-0.45**	0.17	0.54**	0.46*

Significant values are bolded and asterisks indicate

* *P* < 0.05 and

** *P* < 0.01.

Methods details can be found in Table 1.

## Discussion

### Effects of fertilization on foliar, litter and root nutrients

There were no changes in either mass-based or area-based community-wide foliar [N] or [P] two years after initial fertilization. Contrary to our predictions and to results reported in other tropical forests ([16,20,46], we did not observe a community-wide change in foliar or litter [P] with increased P availability, although we did observe an increase in growth and foliar [P] for the most abundant canopy palm (see next section). In Panama, chronic P additions resulted in higher foliar [P] in three out of four tree species—but not lower resorption—after 13 years [20] but other tropical forests have shown foliar [P] responses after two years or less [26,47,48]. The high growth rates and dynamism characteristic of lowland wet forests in our study area [29,37] suggest that P accumulation could have been observed within the timespan of our study. More likely, trees are utilizing extra resources for reproduction [49] or accumulating extra P in other tissues. Fine roots, for example, showed a 12% increase in [P] following P additions. This decoupled response of root and foliar [P] to fertilization has been observed in other studies and can be attributed to fundamental physiological, morphological, and functional differences between leaves and roots [50]. In any case, this high sensitivity of fine roots to P additions shows that root nutrient concentrations were a more refined indicator of soil nutrient status than foliar nutrients [51,52]. More detailed observations are necessary to determine if the root response was ubiquitous or specific for certain functional groups and taxa.

We originally expected to see luxury consumption of N after fertilization but stable levels of [N] were not surprising given that mean foliar [N] at EARTH forest was 2.65% with values up to 5%. These values are at the high end of the global leaf spectrum [53] and are probably close to species-specific physiological and ecological maximums. In fact, it was surprising to observe species-specific increases in foliar [N] after NP additions for the most common tree species (see section below), although the magnitude of the foliar [N] increase was remarkably consistent with responses in other tropical studies (~10%; [15,16,20].

### Influence of tree size class and taxa on responses to fertilization

There were diverse and contrasting results to fertilization between tree size classes and species. Contrary to results obtained in monospecific forests in Hawaii [54], responses to experimental nutrient additions in more diverse forests appear to be driven by the interaction of life history traits and resource availability [20,29,55]. At EARTH Forest, large trees responded to fertilization by increasing their foliar nutrient concentrations but not their stem growth, and small trees responded by increasing their stem growth but not their foliar nutrient concentrations (see [29] and Fig 5). This illustrates a difference in nutrient use strategies among age groups and highlights that nutrient limitation is a dynamic process that can fluctuate for a given tree over time, as physiological and ecological requirements change [17,29,55]. For example, smaller trees may be more light-limited, leading to a response in stem growth, while trees that have reached the canopy may preferentially store extra nutrients in foliage and/or reproduction [39]. As a tree grows, the nutrient requirements change, and so does the response.

Large (> 10 cm DBH) trees of *Pentaclethra*, the dominant tree species in this forest, had approximately 10% higher foliar [N] (but not higher [P]) in the NP treatment, relative to the control. The significant increase in foliar N after fertilization was unexpected given that *Pentaclethra* is capable of fixing atmospheric N_2_ [56], and presumably should not need to sequester this nutrient in N-rich soil, especially when high foliar [N] has been associated with increased levels of herbivory (for example see [57] but see [51,58]). However, *Pentaclethra* flushes leaves and produces flowers and fruits throughout the year, so that fluctuations in foliar and litter nutrients may reflect a combination of phenological demands and soil nutrient availability [49].

The most abundant palm species at EARTH Forest, *Socratea exorrhiza*, was the only taxon (from those observed individually) to show a strong response to fertilization by increasing both RGR and foliar [P] in the P and NP treatments (Fig 5B). This can be interpreted as: (1) from the observed taxa, *Socratea* was the only species clearly limited by P *sensu* [23], or (2) *Socratea* has specific life-history traits that influenced its responsiveness. For example, the response of *Socratea* could have been influenced by its single stem architecture with no branches, relatively high RGR resulting from cell expansion (as opposed to secondary growth), and the formation of dense, superficial root mats, which would be beneficial for fast nutrient uptake after fertilization [59]. We suspect that a combination of both P limitation and life history traits influenced the response of this palm to fertilization.

Interestingly, if *Socratea* was limited by P, as explained by the RGR and foliar P response, we would expect this species to have a high foliar N:P ratio, at least relative to other taxa in the same site. However, the mean foliar N:P ratio for *Socratea* was the lowest among the studied taxa (16.93 ± 0.37). This underscores the supposition that in this system, foliar N:P ratios may be more influenced by inherent species traits than by resource availability [5,17].

### Predicting nutrient limitation of NPP based on plant chemistry

Overall, the tree community at EARTH forest was unable to up-regulate nutrient use after a relatively short-term increase in N and P availability. This observation is illustrated by a lack of community-wide change in foliar [N] and [P], leaf structural changes (SLA or area-based nutrients), stem growth, litterfall production, or root growth index (Table 1). This lack of responsiveness, in combination with relatively high soil nutrient levels and rates of tree growth, suggests that N or P do not strongly limit NPP at EARTH Forest [29] and that other nutrients (for example potassium [55,60], may be important drivers of biogeochemical processes in this forest. Thus—consistent with stoichiometric theory [34]—we expected to observe a community-mean foliar N:P ratio between 14 and16 (mass based). However, the mean foliar N:P ratio was 22.50 (± 0.96), a value traditionally considered indicative of P limitation [5,7,34,35]. This value is higher than mean N:P ratios at a P-limited site in Hawaii (17.23±1.1;[16]) and at Osa Peninsula, a low P forest in the south of Costa Rica (16.4 ± 4.7; [17]). Likely, in sites where nutrient limitation is slightly relaxed, other factors, such as life history traits can become more important controls over tissue nutrient concentrations and the stoichiometric signal of the plant community. Thus, N:P ratios at EARTH forest may reflect the high abundance of legumes (*Pentaclethra* in particular) rather than the nutritional status of the plant community.

Finally, our ability to predict NPP based on plant tissue [N] or [P] was obscured by inconsistent correlations between specific indices of productivity (various measurements of stem growth or litterfall productivity) and leaf or root [N] and [P] (Table 2). Because we did not see a strong response to fertilization, our range of values may have not been enough to observe significant relationships (Table 1). To obtain strong correlations among foliar nutrient concentrations and NPP it is necessary to conduct observations either at a large spatial scale [8,9] or in a low diversity/low nutrient system where responses to nutrient additions overpower natural variability [6,23]. An exception was the strong correlation between root growth and biomass and root [P]. Plots with higher root biomass and growth had lower root [P]. This is opposite of the responses to fertilization observed in montane forests in Hawaii [61] but is not surprising given the multitude of factors that can be driving this relationship. For example, higher nutrient availability can trigger root growth, thus diluting P stored within root tissues.

Collectively, our results support a growing body of evidence stating that nutrient limitation in diverse tropical forests is a complex phenomenon, where different functional groups, size classes, and even plant organs have different nutrient requirements that vary in space and time ([17,28,49,53]. To fully capture the role that nutrient availability plays on plant communities and ecosystems, and to predict how environmental changes will affect nutrient limitation in tropical forests, we recommend conducting a suite of observations and experiments that include—but are not limited to—tissue nutrient concentrations [19].

## Supporting Information

S1 Table Taxa selected. Taxa selected to study the influence of specific taxa on responses to fertilization.
(PDF)

S2 Table MANOVA results for community analysis. Results from repeated measures MANOVAs for foliar, litter and root chemistry.
(PDF)

S3 Table MANOVA results by size. Results from repeated measures MANOVAs for foliar chemistry by tree size class.
(PDF)

S4 Table MANOVA results by taxa. Results from repeated measures MANOVAs for foliar chemistry comparing the six most common taxa (see S1 Table for taxa).
(PDF)

S5 Table Raw data for community analyses. (PDF)

S6 Table Raw data by tree species and size. (PDF)

## References

[1] Melillo JM, McGuire AD, Kicklighter DW, Moore B, Vorosmarty CJ, Schloss AL (1993). Global climate change and terrestrial net primary production. Nature 363: 234–240.

[2] Thornton PE, Lamarque JF, Rosenbloom NA, Mahowald NM (2007). Influence of carbon–nitrogen cycle coupling on land model response to CO2 fertilization and climate variability. GBC 21: GB4018.

[3] Bonan GB, Levis S (2010) Quantifying carbon-nitrogen feedbacks in the Community Land Model (CLM4). Geophys Res Lett 37: L07401.

[4] Koerselmann W, Meuleman AFM (1996) The vegetation N:P ratio: a new tool to detect the nature of nutrient limitation. J Appl Ecol 33:1441–1450.

[5] Güsewell S (2004) N:P ratios in terrestrial plants: variation and functional significance. New Phytol 164:243–266. 10.1111/j.1469-8137.2004.01192.x 33873556

[6] Elser JJ, Bracken ME, Cleland EE, Gruner DS, Harpole WS, Hillebrand H, et al (2007) Global analysis of nitrogen and phosphorus limitation of primary producers in freshwater, marine and terrestrial ecosystems. Ecol Lett 10: 1135–1142. 17922835 10.1111/j.1461-0248.2007.01113.x

[7] Ågren GI (2008) Stoichiometry and nutrition of plant growth in natural communities. Annu Rev Ecol Evol S 39: 153–170.

[8] Cleveland CC, Townsend AR, Taylor P, Alvarez-Clare S., Bustamante M, Chuyong G, et al (2011) Relationships among net primary productivity, nutrients and climate in tropical rain forest: a pan-tropical analysis. Ecol Lett 14: 939–947. 10.1111/j.1461-0248.2011.01658.x 21749602

[9] McGroddy ME, Daufresne T, Hedin LO (2004) Scaling of C:N:P stoichiometry in forests worldwide: Implications of terrestrial Redfield-type ratios. Ecology 85: 2390–2401.

[10] Yuan ZY, Chen YHH, Reich PB (2011) Global-scale latitudinal patterns of plant fine-root nitrogen and phosphorus. Nature Comm 2: 344. 10.1038/ncomms1346 21673665

[11] Reed SC, Vitousek PM, Cleveland CC (2011) Are patterns in nutrient limitation belowground consistent with those aboveground: results from a 4 million year chronosequence. Biogeochemistry 106: 323–336.

[12] Vergutz L, Manzoni S, Porporato A, Novais RF, Jackson RB (2012). Global resorption efficiencies and concentrations of carbon and nutrients in leaves of terrestrial plants. Ecol Monogr 82: 205–20.

[13] Hedin LO (2004) Global Organization of Terrestrial Plant-Nutrient Interactions. P Natl Acad Sci USA 30:10849–10850. 15263081 10.1073/pnas.0404222101 PMC503708

[14] Reich PB, Oleksyn J (2004) Global patterns of plant leaf N and P in relation to temperature and latitude. P Natl Acad Sci USA 101: 11001–11006. 15213326 10.1073/pnas.0403588101 PMC503733

[15] Harrington RA, Fownes JH, Vitousek PM (2001) Production and resource use efficiencies in N- and P-limited tropical forests: A comparison of responses to long-term fertilization. Ecosystems 4: 646–657.

[16] Ostertag R (2010) Foliar nitrogen and phosphorus accumulation responses after fertilization: an example from nutrient-limited Hawaiian forests. Plant Soil 334: 85–98.

[17] Townsend AR, Cleveland CC, Asner GP, Bustamante MMC (2007). Controls over foliar N:P ratios in tropical rain forests. Ecology 88: 107–118. 17489459 10.1890/0012-9658(2007)88[107:cofnri]2.0.co;2

[18] Vitousek PM, Porder S, Houlton BZ, Chadwick OA (2010) Terrestrial phosphorus limitation: mechanisms, implications, and nitrogen-phosphorus interactions. Ecol Appl 20: 5–15. 20349827 10.1890/08-0127.1

[19] Sullivan BW, Alvarez-Clare S, Castle SC, Porder S, Reed SC, Schreeg L (2014). Assessing nutrient limitation in complex forested ecosystems: alternatives to large-scale fertilization experiments. Ecology 95: 668–681. 24804451 10.1890/13-0825.1

[20] Mayor J. R., Wright S. J., & Turner B. L. (2014). Species—specific responses of foliar nutrients to long—term nitrogen and phosphorus additions in a lowland tropical forest. J Ecol 102(1): 36–44.

[21] Hattenschwiler S, Aeschlimann B, Couteaux MM, Roy J, and Bonal D (2008) High variation in foliage and leaf litter chemistry among 45 tree species of a neotropical rainforest community. New Phytol 179: 165–175. 10.1111/j.1469-8137.2008.02438.x 18422903

[22] Andersen KM., Corre MD, Turner BL, Dalling JW (2010) Plant-soil associations in a lower montane tropical forest: physiological acclimation and herbivore-mediate responses to nitrogen addition. Func Ecol 24: 1171–1180.

[23] Chapin FS III, Vitousek PM, Van Cleve K (1986) The nature of nutrient limitation in plant communities. Am Nat 127: 48–58.

[24] van der Ploeg RR, Böhm W, Kirkham MB (1999). On the origin of the theory of mineral nutrition of plants and the law of the minimum. Soil Sci Soc Am J 63:1055–1062.

[25] Vitousek PM, Farrington H (1997) Nutrient limitation and soil development: Experimental test of a biogeochemical theory. Biogeochemistry 37: 63–75.

[26] Tanner EVJ, Vitousek PM, Cuevas E (1998). Experimental investigation of nutrient limitation of forest growth on wet tropical mountains. Ecology 79: 10–22.

[27] Davidson EA, Reis de Carvalho CJ, Figueira AM., Ishida FY, Ometto JPHB, Nardoto GB, et al (2007) Recuperation of nitrogen cycling in Amazonian forests following agricultural abandonment. Nature 447: 995–998. 17581583 10.1038/nature05900

[28] Mirmanto E, Proctor J, Green J, Nagy L Suriantata (1999) Effects of nitrogen and phosphorus fertilization in a lowland evergreen rainforest. Phil. Trans. R. Soc. Lond. 354: 1825–1829. 11605625 10.1098/rstb.1999.0524 PMC1692691

[29] Alvarez-Clare S, Mack MC, Brooks M (2013) A direct test of nitrogen and phosphorus limitation to net primary productivity in a lowland tropical wet forest. Ecology 94: 1540–1551. 23951714 10.1890/12-2128.1

[30] Lambers H, Chapin FS III, Pons TL (1998) Plant Physiological Ecology. Springer-Verlag, New York.

[31] Kobe RK, Lepczyk CA, Iyer M (2005) Resorption efficiency decreases with increasing green leaf nutrients in a global data set. Ecology 86: 2780–2792.

[32] Kerkhoff AJ, Fagan WF, Elser JJ, Enquist BJ (2006) Phylogenetic and functional variation in the scaling of nitrogen and phosphorus in the seed plants. Am Nat 168: E103–E122. 17004214 10.1086/507879

[33] Wood TE, Lawrence D, Clark DA, Chazdon RL (2009) Rain forest nutrient cycling and productivity in response to large-scale litter manipulation. Ecology 90: 109–121. 19294918 10.1890/07-1146.1

[34] Sterner RW, Elser JJ (2002) Ecological stoichiometry: the biology of elements from molecules to the biosphere. Princeton University Press, New Jersey

[35] Ågren GI (2004) The C:N:P stoichiometry of autotrophs—theory and observations. Ecol Lett 7: 185–191.

[36] Sancho F, Mata R, Molina E, Salas R (1990) Estudio de suelos finca de la Escuela de Agricultura de la Región Tropical Húmeda. Reporte para la Escuela de Agricultura de la Región Tropical Húmeda (EARTH), Costa Rica.

[37] McDade LA, Bawa KS, Hespenheide HA, Hartshorn GS (1994) La Selva: ecology and natural history of a neotropical rain forest. The University of Chicago Press.

[38] Hartshorn GS (1983) Plants In: Jansen DH, editor. Costa Rican Natural History. The University of Chicago Press.

[39] Hartshorn GS, Hammel BE (1994) Vegetation types and floristic patterns In: McDade LA, Bawa KS, Hespenheide HA, Hartshorn GS, editors. La Selva: ecology and natural history of a neotropical rain forest. The University of Chicago Press.

[40] Alvarez Clare S (2012) Biological processes influencing nutrient limitation in a lowland tropical wet forest in Costa Rica. PhD Dissertation, University of Florida, Gainesville, FL, USA. Available in: http://ufdc.ufl.edu/UFE0044034. Accessed 2 October, 2014

[41] Clark D. A., Brown S., Kicklighter D. W., Chambers J. Q., Thomlinson J. R., & Ni J. (2001). Measuring net primary production in forests: concepts and field methods. Ecol Appl 11: 356–370.

[42] Cuevas E, Medina E (1988) Nutrient dynamics within Amazonian forests: fine root-growth, nutrient availability and leaf litter decomposition. Oecologia 76: 222–235. 28312200 10.1007/BF00379956

[43] Jones JB, Case BW (1996) Soil testing and plant analysis no. 3 In: Sparks DL, editor. Methods of soil analysis Part 3: chemical methods. Soil Science Society of America, pp 389–415.

[44] Field A (2009) Discovering statistics using SPSS. SAGE Publications Ltd, California.

[45] Zar JH (1996) Biostatistical Analysis. Prentice Hall.

[46] Hidaka A, Kitayama K (2011) Allocation of foliar phosphorus fractions and leaf traits of tropical tree species in response to decreased soil phosphorus availability on Mount Kinabalu, Borneo. J Ecol 99: 849–857.

[47] Herbert DA, Fownes JH (1995). Phosphorus limitation of forest leaf area and net primary production on a highly weathered soil. Biogeochemistry 29: 223–235.

[48] Davidson EA, Reis de Carvalho CJ, Vieira ICG, Figueiredo R, Moutinho P (2004). Nitrogen and phosphorus limitation of biomass growth in a tropical secondary forest. Ecol Appl 14: 150–163.

[49] Tully K., Wood TE, Schwantes AM., Lawrence D (2013) Soil nutrient availability and reproductive effort drive patterns in nutrient resorption in *Pentaclethra macroloba* . Ecology 94: 930–940.

[50] Reich PB, Tjoelker MG, Pregitzer KS, Wright IJ, Oleksyn J (2008) Scaling of respiration to nitrogen in leaves, stems and roots of higher land plants. Ecol Lett 11: 793–801. 10.1111/j.1461-0248.2008.01185.x 18445031

[51] Santiago LS, Wright SJ, Harms KE, Yavitt JB, Korine C, Garcia MN, et al (2012). Tropical tree seedling growth responses to nitrogen, phosphorus and potassium addition. J Ecol 100: 309–316.

[52] Schreeg LA, Santiago LS, Wright SJ, Turner BL (2014). Stem, root, and older leaf N: P ratios are more responsive indicators of soil nutrient availability than new foliage. Ecology, 95: 2062–2068. 25230458 10.1890/13-1671.1

[53] Wright IJ, Reich PB, Westoby M, Ackerly DD, Baruch Z, Bongers F, et al (2004). The worldwide leaf economics spectrum. Nature 428: 821–827. 15103368 10.1038/nature02403

[54] Vitousek PM (2004). Nutrient cycling and limitation: Hawai'i as a model system. Princeton University Press.

[55] Wright SJ, Yavitt JB, Wurzburger N, Turner BL, Tanner EV, Sayer EJ, et al (2011) Potassium, phosphorus or nitrogen limit root allocation, tree growth and litter production in a lowland tropical forest. Ecology 92: 1616–1625. 21905428 10.1890/10-1558.1

[56] Gentry AH 1988 Changes in plant community diversity and floristic composition on environmental and geographical gradients. Ann Mo Bot Gard 75: 1–34.

[57] Huberty AF, Denno RF (2006) Consequences of nitrogen and phosphorus limitation for the performance of two planthoppers with divergent life-history strategies. Oecologia 149: 444–455. 16794833 10.1007/s00442-006-0462-8

[58] Campo J, Dirzo R (2003) Leaf quality and herbivory responses to soil nutrient addition in secondary tropical dry forest of Yucatan, Mexico. J Trop Ecol 19: 525–530.

[59] Henderson A, Galeano G, Bernal R (1995) Field Guide to the Palms of the Americas. Princeton University Press.

[60] Kaspari M, García MN, Harms KE, Santana M, Wright SJ, Yavitt JB (2008) Multiple nutrients limit litterfall and decomposition in a tropical forest. Ecol Lett 11: 35–43. 18021246 10.1111/j.1461-0248.2007.01124.x

[61] Ostertag R (2001) Effects of nitrogen and phosphorus availability on fine-root dynamics in Hawaiian montane forests. Ecology 82: 485–499.

[62] Kuo S (1996) Phosphorus In: Sparks DL, editor. Methods of soil analysis. Part 3: Chemical methods. Soil Sci Soc Am J.

