# Supplementary material for: Do Foliar, Litter, and Root Nitrogen and Phosphorus Concentrations Reflect Nutrient Limitation in a Lowland Tropical Wet Forest?
Source: PLoS One. 2015 Apr 22;10(4):e0123796. doi: 10.1371/journal.pone.0123796 (PMC4406610; doi:10.1371/journal.pone.0123796)
Supplement: S1 Table — Taxa selected to study the influence of specific taxa on responses to fertilization. (PDF) [file pone.0123796.s001.pdf]

Table S1 Taxa selected to study the influence of specific taxa on responses to fertilization

Species	Family	% plots	# trees	Functional properties at EARTH Forest
Dendropanax arboreus	Araliaceae	50	21	Subcanopy tree with soft wood, fast growth, and relatively high light demand.
Goethalsia meiantha	Malvaceae	50	49	Canopy or subcanopy tree with soft wood, fast growth, and relatively high light demand. Characteristic of disturbed areas.
Inga	Mimosaceae	70	25	Seven species included[1]. Canopy or subcanopy trees with soft or semi hard wood, and mostly shade tolerant. Can fix Nitrogen.
Pentaclethra macroloba	Mimosaceae	100	91	Canopy tree with semi hard wood, medium growth, and shade tolerant. Most abundant tree at EARTH forest. Can fix Nitrogen.
Protium	Burseraceae	75	41	Four species included[2]. Subcanopy trees (at EARTH forest) with relatively slow growth, and shade tolerant. Resinous compounds in leaves, stems, and fruits.
Socratea exohrriza	Arecaceae	100	154	Canopy/subcanopy palm with relatively fast growth and shade tolerant. Conspicuous stilt roots.

Shown is the percent of the plots in which a taxa were present (n = 24 plots) and the total number of trees measured (all trees were >4 cm DBH). All taxa had at least one individual in each treatment.

Source McDade et al. (1994) and O. Vargas (personal communication).

[1] *I. alba, I. leocalycina, I. pezizifera, I. sapindioides, I. thiboudiana, I. umbilifera, I. venusta*

[2] *P. confusum, P. panamense, P. pittieri, P. ravenii*
