# Supplementary material for: Do Foliar, Litter, and Root Nitrogen and Phosphorus Concentrations Reflect Nutrient Limitation in a Lowland Tropical Wet Forest?
Source: PLoS One. 2015 Apr 22;10(4):e0123796. doi: 10.1371/journal.pone.0123796 (PMC4406610; doi:10.1371/journal.pone.0123796)
Supplement: S2 Table — Results from repeated measures MANOVAs for foliar, litter and root chemistry. (PDF) [file pone.0123796.s002.pdf]

Table S2 Results from repeated measures MANOVAs for foliar, litter and root chemistry

	$df_{n,d}$	*F*	*P* value
Foliar %N			
Treatment	3,15	1014	0.41
**Block**	**5,15**	**5.57**	**<0.01***
Time	2,14	0.17	0.85
Time*treatment	6,28	1.73	0.15
Time*block	10,28	0.78	0.65
Foliar P (mg g^{-1})			
Treatment	3,15	0.42	0.74
**Block**	**5,15**	**3.79**	**0.02***
**Time**	**2,14**	**3.94**	**0.04***
Time*treatment	6,28	1.36	0.27
Time*block	10,28	1.06	0.43
Foliar N:P			
Treatment	3,15	0.47	0.71
Block	5,15	0.57	0.72
Time	2,14	0.27	0.77
Time*treatment	6,28	0.87	0.91
Time*block	10,28	0.88	0.99
Litterfall %N			
**Treatment**	**3,15**	**2.43**	**0.11**[1]
Block	5,15	2.16	0.11
**Time**	**2,14**	**15.13**	**<0.01***
Time*treatment	6,28	1.97	0.10
Time*block	10,28	01.16	0.36
Litterfall P (mg g^{-1})			
Treatment	3,15	2.66	0.09
**Block**	**5,15**	**2.98**	**0.04***
**Time**	**2,14**	**11.68**	**<0.01***
Time*treatment	6,28	0.78	0.72
Time*block	10,28	0.56	0.83
Litterfall N:P			
Treatment	3,15	1.23	0.34
Block	5,15	0.63	0.68
**Time**	**2,14**	**8.05**	**<0.01***
Time*treatment	6,28	0.30	0.93
Time*block	10,28	0.85	0.59
Root %N			
Treatment	3,15	0.81	0.51
Block	5,15	1.14	0.38
**Time**	**2,14**	**5.56**	**0.02***
Time*treatment	6,28	1.47	0.22
Time*block	10,28	1.15	0.36
Root P (mg g^{-1})			
Treatment	3,14	2.29	0.12

**Block**	**5,14**	**3.35**	**0.03***
**Time**	**2,13**	**31.9**	**<0.01***
**Time*treatment**	**6,26**	**4.32**	**<0.01***
Time*block	10,26	2.11	0.06
Root N:P			
Treatment	3,15	0.93	0.45
**Block**	**5,15**	**3.20**	**0.04***
**Time**	**2,14**	**11.38**	**<0.01***
Time*treatment	6,28	1.31	0.29
Time*block	10,28	1.34	0.26

F-values for treatment, block and time were obtained from exact tests but time*treatment and time*block interactions are F-value approximations resulting from Wilk’s lambda multivariate tests. In these cases degrees of freedom (df) are approximated as well. Significant effects are signaled with an asterisk.

[1] Treatment effect becomes significant if non-significant block effect is removed from analysis (Time*treatment $F_{6,38} = 2.37$ $P = 0.04$).
