# Supplementary material for: Do Foliar, Litter, and Root Nitrogen and Phosphorus Concentrations Reflect Nutrient Limitation in a Lowland Tropical Wet Forest?
Source: PLoS One. 2015 Apr 22;10(4):e0123796. doi: 10.1371/journal.pone.0123796 (PMC4406610; doi:10.1371/journal.pone.0123796)
Supplement: S3 Table — Results from repeated measures MANOVAs for foliar chemistry by tree size class. (PDF) [file pone.0123796.s003.pdf]

Table S3 Results from repeated measures MANOVAs for foliar chemistry by tree size class

	$df_{n,d}$	*F*	*P* value
Foliar %N			
5-10 cm			
Treatment	3,15	2.60	0.09
Block	5,15	0.81	0.56
Time	2,14	0.80	0.47
Time*treatment	6,28	0.28	0.94
Time*block	10,28	0.57	0.82
> 10 cm			
**Treatment**	**3,15**	**3.35**	**0.04***
**Block**	**5,15**	**8.22**	**<0.01***
Time	2,14	0.52	0.61
**Time*treatment**	**6,28**	**3.42**	**0.01***
Time*block	10,28	0.94	0.51
Foliar P (mg g^{-1})			
5-10 cm			
**Treatment**	**3,15**	**4.02**	**0.03***
Block	5,15	2.58	0.07
Time	2,14	2.46	0.12
Time*treatment	6,28	2.25	0.07
Time*block	10,28	1.16	0.36
> 10 cm			
Treatment	3,15	0.72	0.56
Block	5,15	2.35	0.09
Time	2,14	1.66	0.23
Time*treatment	6,28	0.50	0.80
Time*block	10,28	0.65	0.76
Foliar N:P			
5-10 cm			
Treatment	3,15	0.45	0.72
Block	5,15	0.93	0.49
Time	2,14	0.52	0.61
Time*treatment	6,28	0.59	0.74
Time*block	10,28	0.48	0.89
> 10 cm			
Treatment	3,15	0.53	0.67
Block	5,15	0.90	0.51
Time	2,14	1.79	0.20
Time*treatment	6,28	0.23	0.96
Time*block	10,28	0.40	0.94

F-values for treatment, block and time were obtained from exact tests but time*treatment and time*block interactions are F-value approximations resulting from Wilk's lambda multivariate tests. In these cases degrees of freedom (df) are approximated as well. Significant effects are signaled with an asterisk.
