# Supplementary material for: Do Foliar, Litter, and Root Nitrogen and Phosphorus Concentrations Reflect Nutrient Limitation in a Lowland Tropical Wet Forest?
Source: PLoS One. 2015 Apr 22;10(4):e0123796. doi: 10.1371/journal.pone.0123796 (PMC4406610; doi:10.1371/journal.pone.0123796)
Supplement: S4 Table — Results from repeated measures MANOVAs for foliar chemistry comparing the six most common taxa (see S1 Table for taxa). (PDF) [file pone.0123796.s004.pdf]

Table S4 Results from repeated measures MANOVAs for foliar chemistry comparing the six most common taxa (see Table S1 for taxa)

	$df_{n,d}$	F	Probability
Foliar %N			
**Taxa**	**5,107**	**78.50**	**< 0.01***
Time	2,106	0.22	0.81
Time*taxa	10,212	1.82	0.06
Foliar P (mg g^{-1})			
**Taxa**	**5,99**	**5.68**	**< 0.01***
Time	2,98	2.78	0.07
**Time*taxa**	**10,196**	**2.98**	**< 0.01***
Foliar N:P ratios			
**Taxa**	**5,98**	**52.67**	**< 0.01***
**Time**	**2,97**	**3.41**	**0.04***
**Time*taxa**	**10,194**	**2.70**	**< 0.01***

F-values for taxa and time were obtained from exact tests but time*taxa interactions are F-value approximations resulting from Wilk's lambda multivariate tests. In these cases degrees of freedom (df) are approximated as well. Significant effects are signaled with an asterisk.
