# Supplementary material for: Do Foliar, Litter, and Root Nitrogen and Phosphorus Concentrations Reflect Nutrient Limitation in a Lowland Tropical Wet Forest?
Source: PLoS One. 2015 Apr 22;10(4):e0123796. doi: 10.1371/journal.pone.0123796 (PMC4406610; doi:10.1371/journal.pone.0123796)
Supplement: S5 Table — (PDF) [file pone.0123796.s005.pdf]

Table S5. N and P concentration data by plot.

Plot	Block	Treatment	Foliar %N PreFert	Foliar %N 1yr	Foliar %N 2yrs	Foliar P (mg/g) PreFert	Foliar P (mg/g) 1yr	Foliar P (mg/g) 2yrs	root%N PreFert	root %N 1yr	root %N 2yrs	root P(mg/g) PreFert	root P(mg/g) 1yr
1	1	N	2.42	2.77	2.54	1.19	1.28	1.24	1.50	1.51	1.57	0.46	0.41
2	1	C	2.41	2.41	2.58	1.21	1.15	1.21	1.58	1.80	1.67	0.44	0.42
3	1	NP	2.66	2.54	2.49	1.28	1.19	1.17	1.53	1.62	1.60	0.43	0.42
4	1	P	2.70	2.74	2.44	1.22	1.30	1.11	1.56	1.51	1.52	0.44	0.36
5	2	NP	2.66	2.72	2.67	1.26	1.18	1.26	1.17	1.84	1.71	0.38	0.48
6	2	P	2.62	2.53	2.51	1.14	1.13	1.19	1.72	1.51	1.76	0.48	0.40
7	2	C	2.63	2.52	2.48	1.12	1.07	1.14	1.54	1.46	1.86	0.41	0.39
8	3	C	2.72	2.37	2.60	1.28	1.32	1.20	1.54	1.72	1.60	0.44	0.44
9	3	P	2.46	2.39	2.51	1.15	1.28	1.18	1.66	1.77	1.71	0.41	0.40
10	3	N	2.52	2.74	2.69	1.45	1.29	1.20	1.85	1.55	1.42	0.53	0.43
11	3	NP	2.37	2.52	2.70	1.29	1.36	1.34	1.62	1.55	1.90	0.38	0.52
12	2	N	2.83	2.90	2.86	1.12	1.22	1.10	1.80	1.83	1.97	0.48	0.43
13	4	N	2.81	2.87	2.69	1.30	1.30	1.26	1.34	1.27	1.53	0.40	0.39
14	4	C	2.61	2.56	2.73	1.24	1.09	1.28	1.53	1.94	1.85	0.40	0.49
15	4	P	2.72	2.77	2.52	1.27	1.29	1.23	1.20	1.39	1.44	0.38	0.48
16	5	P	2.89	2.96	3.09	1.48	1.34	1.36	1.44	1.34	1.63	0.51	0.43
17	5	N	2.69	2.85	2.69	1.26	1.33	1.20	1.29	1.36	1.62	0.43	0.49
18	5	NP	2.91	2.89	2.91	1.17	1.21	1.10	1.40	1.97	1.85	0.42	0.48
19	5	C	2.91	2.78	2.83	1.32	1.20	1.34	1.56	1.79	1.59	0.55	0.43
20	4	NP	2.69	2.71	2.67	1.22	1.25	1.25	1.54	1.61	1.59	0.42	0.47
21	6	P	2.85	2.89	2.88	1.37	1.48	1.29	1.78	1.79	1.77	0.57	0.54
22	6	N	2.76	2.59	2.69	1.32	1.31	1.20	1.83	1.48	1.62	0.55	0.41
23	6	NP	2.58	2.67	2.61	1.26	1.38	1.32	1.48	1.44	1.86	0.51	0.44
24	6	C	2.68	2.69	2.69	1.32	1.40	1.26	1.84	1.58	1.71	0.51	0.44

Table S5. N and P concentration data by plot.

Plot	Block	Treatment	root P(mg/g) 2yrs	L itterfall%N PreFert	Litterfall %N 1yr	Litterfall %N 2yrs	LitterfallP(mg/g) PreFert	Litterfall P(mg/g) 1yr	Litterfall P(mg/g) 2yrs
1	1	N	0.48	2.05	2.42	2.50	0.67	0.98	1.20
2	1	C	0.49	1.89	1.85	2.04	0.62	0.65	0.83
3	1	NP	0.56	2.09	2.05	2.24	0.96	0.85	1.01
4	1	P	0.54	1.91	2.15	2.22	0.60	0.79	0.72
5	2	NP	0.47	2.03	2.41	2.63	0.80	1.03	1.12
6	2	P	0.64	2.28	1.88	2.32	0.83	0.62	0.76
7	2	C	0.47	1.91	1.83	2.05	0.94	0.80	0.90
8	3	C	0.44	1.97	1.93	2.20	0.82	0.72	1.04
9	3	P	0.42	2.05	1.94	2.12	0.57	0.80	0.80
10	3	N	0.48	2.09	2.18	2.37	0.83	0.90	1.08
11	3	NP	0.60	2.21	2.43	2.62	0.71	0.70	0.81
12	2	N	0.42	2.26	2.12	2.69	0.68	0.74	0.97
13	4	N	0.55	2.47	2.86	2.68	1.14	1.13	1.05
14	4	C	0.54	2.33	2.64	2.57	0.90	1.02	1.01
15	4	P	0.73	2.60	2.26	2.42	0.99	0.88	0.80
16	5	P	0.70	2.44	2.38	2.11	0.89	0.98	0.91
17	5	N	0.48	1.93	2.39	2.56	1.18	0.94	1.08
18	5	NP	0.71	2.41	2.73	2.85	1.12	0.95	1.16
19	5	C	0.97	2.17	2.61	2.46	0.96	0.93	1.18
20	4	NP	0.57	2.03	1.90	2.27	0.74	0.62	1.00
21	6	P	0.83	2.24	2.32	2.07	1.04	0.67	1.09
22	6	N	0.48	2.35	2.39	2.56	0.85	0.94	1.08
23	6	NP	0.64	2.38	2.51	2.40	0.84	1.05	1.07
24	6	C	0.58	1.84	2.06	2.31	0.73	0.86	0.95
