# Supplementary material for: Do Foliar, Litter, and Root Nitrogen and Phosphorus Concentrations Reflect Nutrient Limitation in a Lowland Tropical Wet Forest?
Source: PLoS One. 2015 Apr 22;10(4):e0123796. doi: 10.1371/journal.pone.0123796 (PMC4406610; doi:10.1371/journal.pone.0123796)
Supplement: S6 Table — (PDF) [file pone.0123796.s006.pdf]

Plot	treatment	block	size	Species Code	Genus	species	Foliar P (mg/g) PreFert	Foliar P (mg/g) 1yr	Foliar P (mg/g) 2yrs	Foliar %N PreFert	Foliar %N 1yr	Foliar %N 2yrs
11	NP	3	large	ApeMem	Apeiba	membranacea	1.52	1.61	1.45	2.32	3.12	3.34
13	N	4	large	ApeMem	Apeiba	membranacea	1.41	1.42	1.40	2.92	2.97	2.47
16	P	5	large	ApeMem	Apeiba	membranacea	1.57	0.89	1.66	3.14	2.80	2.91
16	P	5	small	ApeMem	Apeiba	membranacea	1.47		1.51	2.84		3.42
17	N	5	small	ApeMem	Apeiba	membranacea	1.17	1.58		2.50	2.80	
19	C	5	large	ApeMem	Apeiba	membranacea	1.63	0.92	1.77	3.06	3.07	3.09
21	P	6	large	ApeMem	Apeiba	membranacea	1.36			2.81		
22	N	6	small	ApeMem	Apeiba	membranacea	1.70			3.38		
23	NP	6	large	ApeMem	Apeiba	membranacea	1.53	1.21	1.17	2.71	2.94	2.93
7	C	2	large	AstAla	Astrocarium	alatum	1.42	0.74	1.22	2.15	1.76	2.01
1	N	1	small	CapPit	Capparis	pittieri	1.12	1.26	1.21	2.82	3.22	2.87
2	C	1	large	CapPit	Capparis	pittieri	1.18	1.37	1.07	2.26	2.66	2.79
2	C	1	small	CapPit	Capparis	pittieri	0.80	0.95	1.16	2.79	2.96	2.97
3	NP	1	large	CapPit	Capparis	pittieri	1.24	1.09	0.83	2.77	3.08	2.73
3	NP	1	small	CapPit	Capparis	pittieri	0.89	1.92	1.06	2.86	3.11	2.68
4	P	1	large	CapPit	Capparis	pittieri		1.12	0.95		2.81	2.60
4	P	1	large	CapPit	Capparis	pittieri	1.17	1.27	0.88	3.08	2.92	2.64
4	P	1	small	CapPit	Capparis	pittieri	1.03		1.01	3.06		2.88
5	NP	2	small	CapPit	Capparis	pittieri	1.63	0.99	1.32	3.33	2.95	3.22
9	P	3	large	CapPit	Capparis	pittieri	0.79	1.06	0.79	2.37	2.53	2.45
9	P	3	small	CapPit	Capparis	pittieri	1.45	1.80	1.07	2.76	3.06	2.90
20	NP	4	small	CapPit	Capparis	pittieri	1.05	0.83	1.13	2.85	2.81	2.72
24	C	6	small	CapPit	Capparis	pittieri	0.79	0.89	1.34	2.13	2.50	2.52
19	C	5	small	CasArb	Cassearia	arborea	1.20	1.17		3.07	3.09	
20	NP	4	small	CasArb	Cassearia	arborea		1.08			3.03	
21	P	6	small	CasArb	Cassearia	arborea			1.00			2.97
7	C	2	small	CassipuEllip	Cassipurea	elliptica	1.09	1.30	1.11	3.56	3.55	3.34
18	NP	5	large	ChrVen	Chrysophyllum	venezuelanens	1.21	1.47	1.06	5.29	5.37	5.17
14	C	4	small	CorBic	Cordia	bicolor	1.42		1.67	2.57		2.30
17	N	5	small	CorBic	Cordia	bicolor	1.22	1.38		2.90	3.35	
22	N	6	large	CorBic	Cordia	bicolor	0.86	1.59		2.61	2.45	

21	P	6	small	CorLuc	Cordia	lucida	1.24	1.42	1.39	2.94	3.52	3.22
13	N	4	small	CouHon	Coussarea	hondensis	1.15	0.99	1.09	2.62	3.37	2.73
20	NP	4	small	CouHon	Coussarea	hondensis			1.18			3.03
3	NP	1	large	CroSch	Croton	schiedeanus	1.51	1.28		3.16	3.29	
3	NP	1	small	CroSch	Croton	schiedeanus	1.49			2.94		
4	P	1	small	CroSch	Croton	schiedeanus	1.56	1.33	1.19	2.70	2.86	
6	P	2	small	CroSch	Croton	schiedeanus	1.42	1.08	1.50	3.04	2.92	2.66
8	C	3	small	CroSch	Croton	schiedeanus	1.54	1.15	1.44	3.10	2.83	2.98
10	N	3	small	CroSch	Croton	schiedeanus	1.80	1.32	1.44	3.08	3.16	3.30
13	N	4	small	CroSch	Croton	schiedeanus	1.25	1.48	1.39	2.85	3.28	3.06
14	C	4	small	CroSch	Croton	schiedeanus			1.19			3.04
15	P	4	small	CroSch	Croton	schiedeanus	1.30	1.26	1.35	3.08	3.60	2.97
16	P	5	small	CroSch	Croton	schiedeanus	1.01		1.36	2.63		2.99
21	P	6	small	CroSch	Croton	schiedeanus	1.36	1.84	1.24	3.44	3.29	3.32
22	N	6	large	CroSch	Croton	schiedeanus	1.63	1.23		3.04	3.24	
22	N	6	small	CroSch	Croton	schiedeanus	1.53	1.47		2.73	2.98	
1	N	1	large	DenArb	Dendropanax	arboreus	1.21	1.63	1.21	2.34	3.00	2.37
3	NP	1	large	DenArb	Dendropanax	arboreus			1.52			2.50
4	P	1	large	DenArb	Dendropanax	arboreus	1.42	1.02	0.88	2.36	2.17	1.96
5	NP	2	large	DenArb	Dendropanax	arboreus		1.02	1.13		2.23	2.31
5	NP	2	small	DenArb	Dendropanax	arboreus	1.17	1.03	1.15	2.53	2.56	2.06
10	N	3	large	DenArb	Dendropanax	arboreus	1.20	1.59	1.06	2.46	2.52	2.47
12	N	2	large	DenArb	Dendropanax	arboreus	1.17	0.98	0.97	2.41	2.73	1.88
12	N	2	small	DenArb	Dendropanax	arboreus	1.62	1.35	1.17	2.39	2.69	2.41
15	P	4	large	DenArb	Dendropanax	arboreus	1.27	1.00	1.10	2.50	2.35	2.78
15	P	4	small	DenArb	Dendropanax	arboreus	1.07	1.08	1.10	2.43	2.25	2.22
17	N	5	large	DenArb	Dendropanax	arboreus	1.16	1.26		2.28	2.53	
18	NP	5	small	DenArb	Dendropanax	arboreus	0.96	0.94	1.27	2.41	2.28	2.61
19	C	5	large	DenArb	Dendropanax	arboreus	1.48	1.34	1.16	2.33	1.72	1.93
20	NP	4	small	DenArb	Dendropanax	arboreus	1.03	1.09		2.15	2.30	
1	N	1	large	DypPan	Dypterix	panamensis			1.70		2.26	2.80
7	C	2	small	FarPar	Faramea	parvibractea	0.75	0.93	0.76	2.38	2.20	2.17
11	NP	3	large	GoeMei	Goethalsia	Meiantha			1.52		2.39	2.60
13	N	4	large	GoeMei	Goethalsia	Meiantha	1.53	1.84	1.56	3.40	3.29	3.58

14	C	4	large	GoeMei	Goethalsia	Meiantha			1.53			3.30
15	P	4	large	GoeMei	Goethalsia	Meiantha	1.65	1.49	1.54	3.42	3.31	2.90
16	P	5	large	GoeMei	Goethalsia	Meiantha	1.96	1.27	1.72	3.63	4.20	3.83
16	P	5	small	GoeMei	Goethalsia	meiantha	1.71		0.98	2.91		
17	N	5	small	GoeMei	Goethalsia	meiantha	1.53	1.45		3.04	3.19	
18	NP	5	large	GoeMei	Goethalsia	Meiantha	1.88	1.17	1.40	3.25	3.10	2.22
19	C	5	large	GoeMei	Goethalsia	Meiantha		1.68	1.95			3.39
20	NP	4	large	GoeMei	Goethalsia	Meiantha	1.68	0.95	1.56	3.44	3.46	3.31
20	NP	4	small	GoeMei	Goethalsia	meiantha	1.63			2.69		
21	P	6	large	GoeMei	Goethalsia	Meiantha		1.41	1.41		2.92	3.24
23	NP	6	large	GoeMei	Goethalsia	Meiantha	1.51	1.43	1.74	3.23	3.61	3.24
24	C	6	large	GoeMei	Goethalsia	Meiantha			1.46			3.20
5	NP	2	large	GuaGui	Guarea	guidonia		1.81	1.74		2.83	3.41
21	P	6	small	GuaGui	Guarea	guidonia	1.75	1.89		2.89	3.16	
19	C	5	small	GuarSp	Guarea	Sp.	1.90	1.50		3.92	3.77	3.87
23	NP	6	small	Guateria S	Guateria	Sp.	0.92	1.07	0.94	2.29	2.30	1.99
21	P	6	small	Heisteria s	Heisteria	Sp.			1.39			3.26
2	C	1	small	IngAlb	Inga	alba	1.15			2.94		
6	P	2	large	IngAlb	Inga	alba	1.64	1.32	1.29	3.91	3.50	3.42
7	C	2	large	IngAlb	Inga	alba	0.76	0.74	0.84	2.69	2.74	2.59
12	N	2	large	IngAlb	Inga	alba	1.16	1.06	1.19	3.88	4.25	3.71
24	C	6	large	IngAlb	Inga	alba	1.88	1.67	1.93	3.29	3.41	3.61
19	C	5	large	IngLeu	Inga	leucalycina	1.45	1.08	1.10	3.64	3.33	3.45
4	P	1	small	IngPez	Inga	pezizifera	1.62	2.19		3.53	4.31	
5	NP	2	large	IngPez	Inga	pezizifera	1.49	1.22	1.15	3.94	3.50	3.63
12	N	2	small	IngPez	Inga	pezizifera	0.84	1.19		3.58	3.38	4.57
13	N	4	small	IngSap	Inga	sapindioides	1.60			3.88		
15	P	4	large	IngSp	Inga	Sp.	1.13	1.52	1.18	3.09	4.06	2.86
10	N	3	large	IngThi	Inga	thiboudiana	0.93	1.09	1.01	2.86	3.02	3.13
14	C	4	small	IngThi	Inga	thiboudiana	1.06	0.69	1.22	2.77	2.63	3.45
17	N	5	large	IngThi	Inga	Thiboudiana	1.07	1.18		2.88	2.99	
21	P	6	large	IngThi	Inga	thiboudiana	1.08	1.38	0.92	2.93	3.04	3.05
22	N	6	large	IngUmb	Inga	umbilifera	1.39	0.82		3.52	3.07	
6	P	2	small	IngVen	Inga	venusta	0.81	1.10	1.02	2.52	2.82	2.70

10	N	3	small	IngVen	Inga	venusta	1.44	0.87	0.96	2.79	2.76	2.86
16	P	5	large	IngVen	Inga	venusta	1.56	1.12	1.13	3.18	2.85	3.15
20	NP	4	large	JacCop	Jacaranda	copaia	1.45	1.24	1.08	3.14	2.95	3.05
11	NP	3	small	MicAff	Miconia	affinis	0.96	1.18	1.11	2.56	2.83	2.84
12	N	2	small	MicCro	Micropholis	crotonoides	0.67	1.50	0.89	3.30	3.79	3.39
18	NP	5	small	MicCro	Micropholis	crotonoides	0.76	0.71	0.63	3.15	3.12	3.15
11	NP	3	small	MicMutis	Miconia	Sp.	1.01	1.13	0.97	2.05	2.10	2.13
24	C	6	small	Myrcia Sp	Myrcia	Sp.	1.11			2.85		
4	P	1	small	NauNag	Nauclopsis	naga			1.18			2.06
20	NP	4	large	NauNag	Nauclopsis	naga	0.95	1.85	1.02	2.31	2.19	2.13
19	C	5	small	NecMem	Nectandra	membranacea	1.12	1.04	1.18	3.26	2.85	2.91
24	C	6	large	NecRet	Nectandra	reticulata	2.16	2.03	1.46	2.82	3.09	3.46
8	C	3	small	OcoLae	Ocotea	leucoxylon			0.98			2.70
23	NP	6	large	OcoLae	Ocotea	laetevirens	0.90	1.97	0.96	2.87	3.07	2.91
15	P	4	small	OcoNov	Ocotea	novogranatensis			0.98			1.81
7	C	2	small	OcoSp	Ocotea	Sp.	1.11	1.29	1.13	3.24	2.88	2.77
1	N	1	large	PenMac	Pentaclethra	macroloba		1.17	1.21	2.65	2.71	3.11
1	N	1	small	PenMac	Pentaclethra	macroloba		1.25	1.01		3.48	2.60
2	C	1	large	PenMac	Pentaclethra	macroloba	1.26	1.10	1.22	2.89	2.96	2.69
2	C	1	large	PenMac	Pentaclethra	macroloba		1.33	0.99			2.52
3	NP	1	large	PenMac	Pentaclethra	macroloba		1.22	1.18	3.16	2.78	3.53
4	P	1	large	PenMac	Pentaclethra	macroloba		0.95	1.02		2.38	2.52
4	P	1	large	PenMac	Pentaclethra	macroloba		1.54			2.99	
5	NP	2	large	PenMac	Pentaclethra	macroloba	1.07	1.37	1.16	2.79	3.10	3.06
5	NP	2	small	PenMac	Pentaclethra	macroloba	1.12	1.16	1.08	2.76	3.22	2.94
6	P	2	large	PenMac	Pentaclethra	macroloba		0.97	0.99		2.44	2.72
6	P	2	large	PenMac	Pentaclethra	macroloba	1.09	1.14	1.07	3.13	2.52	2.86
6	P	2	small	PenMac	Pentaclethra	macroloba	1.40	1.10	1.26	2.83	2.66	2.64
7	C	2	large	PenMac	Pentaclethra	macroloba	1.22	1.35	1.23	3.02	3.23	3.30
7	C	2	large	PenMac	Pentaclethra	macroloba		1.19			2.85	
8	C	3	large	PenMac	Pentaclethra	macroloba	1.14	1.12	1.13	2.85	2.27	2.83
8	C	3	small	PenMac	Pentaclethra	macroloba	1.16	1.17	1.10	2.95	2.72	2.55
9	P	3	large	PenMac	Pentaclethra	macroloba	1.10	0.90	1.13	2.92	2.65	2.33
10	N	3	large	PenMac	Pentaclethra	macroloba	1.05	1.26	1.09	2.96	3.11	2.58

10	N	3	small	PenMac	Pentaclethra	macroloba		1.20	1.11		3.31	2.82
11	NP	3	large	PenMac	Pentaclethra	macroloba	1.57	1.41		2.93	2.94	
11	NP	3	large	PenMac	Pentaclethra	macroloba			1.22			3.06
11	NP	3	large	PenMac	Pentaclethra	macroloba						3.29
11	NP	3	small	PenMac	Pentaclethra	macroloba	1.25	1.18	1.14	2.91	2.85	3.04
12	N	2	large	PenMac	Pentaclethra	macroloba	1.12	1.16	1.16	2.76		2.92
12	N	2	small	PenMac	Pentaclethra	macroloba		1.36	1.05			2.75
13	N	4	large	PenMac	Pentaclethra	macroloba		1.04	1.15		3.21	2.96
13	N	4	small	PenMac	Pentaclethra	macroloba			1.19			2.71
13	N	4	small	PenMac	Pentaclethra	macroloba	1.06	1.00	1.13	2.91	2.68	2.93
14	C	4	large	PenMac	Pentaclethra	macroloba	1.04	1.17	1.05	2.93	2.73	2.73
14	C	4	small	PenMac	Pentaclethra	macroloba	1.27	1.38		2.66	2.72	
14	C	4	small	PenMac	Pentaclethra	macroloba		1.31	1.21		3.10	2.58
15	P	4	large	PenMac	Pentaclethra	macroloba	1.02	1.49	1.26	2.68	2.37	2.90
15	P	4	large	PenMac	Pentaclethra	macroloba		1.20				2.46
15	P	4	small	PenMac	Pentaclethra	macroloba	1.36	1.14	1.16	3.54	3.02	2.77
16	P	5	large	PenMac	Pentaclethra	macroloba			1.30			3.45
16	P	5	large	PenMac	Pentaclethra	macroloba	1.40	1.87	1.21	3.26	2.89	3.19
16	P	5	small	PenMac	Pentaclethra	macroloba	1.44		1.31	3.18		3.51
17	N	5	large	PenMac	Pentaclethra	macroloba	1.23	1.89		3.15	3.46	
17	N	5	small	PenMac	Pentaclethra	macroloba	1.16	1.17		2.93	2.49	
18	NP	5	large	PenMac	Pentaclethra	macroloba	1.20	1.95	1.32	2.74	2.75	3.18
18	NP	5	small	PenMac	Pentaclethra	macroloba	1.17	0.98	1.13	2.80	2.90	2.53
19	C	5	large	PenMac	Pentaclethra	macroloba	1.27	0.94	1.24	2.73	2.68	2.81
19	C	5	small	PenMac	Pentaclethra	macroloba	1.12	1.25	1.11	2.72	2.77	2.52
20	NP	4	large	PenMac	Pentaclethra	macroloba	1.41	1.47	1.09	2.98	2.59	2.93
20	NP	4	small	PenMac	Pentaclethra	macroloba			1.20			2.96
21	P	6	large	PenMac	Pentaclethra	macroloba			1.00			2.99
21	P	6	large	PenMac	Pentaclethra	macroloba	1.33	1.10	1.13	2.99	2.62	2.57
21	P	6	small	PenMac	Pentaclethra	macroloba		1.14	1.42			2.69
22	N	6	large	PenMac	Pentaclethra	macroloba	1.24	1.48		2.92	2.91	
22	N	6	large	PenMac	Pentaclethra	macroloba		1.33				
23	NP	6	large	PenMac	Pentaclethra	macroloba	1.19	1.25		3.02	3.07	3.20
23	NP	6	small	PenMac	Pentaclethra	macroloba		1.27			3.27	2.94

24	C	6	large	PenMac	Pentaclethra	macroloba			1.16			3.10
24	C	6	large	PenMac	Pentaclethra	macroloba	1.18	1.67	1.23	3.21	3.45	3.20
18	NP	5	large	Poucal	Pouteria	calistophylla	1.00	1.43	0.81	2.56		2.29
1	N	1	small	ProCon	Protium	confusum	1.05	1.04	1.08	1.86	2.10	1.85
2	C	1	small	ProCon	Protium	confusum	1.28	1.03	1.13	1.99	1.96	1.97
3	NP	1	large	ProCon	Protium	confusum			1.11			2.15
3	NP	1	large	ProCon	Protium	confusum	1.23	1.31	1.13	2.08	2.07	2.17
4	P	1	large	ProCon	Protium	confusum	0.97	1.31	0.97	2.17	2.31	2.04
5	NP	2	large	ProCon	Protium	confusum	0.98		1.11	2.06		1.94
5	NP	2	small	ProCon	Protium	confusum	1.37	0.94	0.95	2.12	1.86	2.09
6	P	2	small	ProCon	Protium	confusum	0.99	1.00	1.04	1.87	1.82	2.04
8	C	3	small	ProCon	Protium	confusum	1.35	1.61	1.20	2.19	2.23	1.96
9	P	3	small	ProCon	Protium	confusum	0.94	1.06	1.24	2.21	2.11	2.38
11	NP	3	small	ProCon	Protium	confusum	1.64	1.98	1.80	2.24	2.65	2.48
14	C	4	small	ProCon	Protium	confusum	1.15	0.90		2.01	1.97	
15	P	4	small	ProCon	Protium	confusum	1.23	1.45	1.04	2.04	2.18	2.05
22	N	6	small	ProCon	Protium	confusum	1.14	0.88		2.20	1.99	
24	C	6	small	ProCon	Protium	confusum	1.32	0.99	0.87	2.03	2.14	1.93
7	C	2	large	ProPan	Protium	panamensis	1.13	1.11	1.29	2.07	2.12	2.10
8	C	3	large	ProPan	Protium	panamensis	1.36	1.77	1.22	2.28	1.81	2.17
9	P	3	large	ProPan	Protium	panamensis	1.25	1.20	1.35	2.13	2.18	2.40
9	P	3	small	ProPan	Protium	panamense	1.45	1.52	1.10	2.40	2.15	3.38
10	N	3	large	ProPan	Protium	panamensis	1.44	1.42	1.38	2.19	2.32	2.30
10	N	3	small	ProPan	Protium	panamense	2.04		1.35	2.35		2.67
11	NP	3	large	ProPan	Protium	panamense	1.27	1.24		2.25	2.24	
14	C	4	large	ProPan	Protium	panamensis	1.26	1.09	1.09	2.22	2.27	2.38
23	NP	6	small	ProPan	Protium	panamense	1.51	1.80	1.71	2.31	2.05	2.40
1	N	1	large	ProPit	Protium	pittieri	1.22	1.32	1.21	2.03	2.61	2.24
2	C	1	large	ProPit	Protium	pittieri	1.26	1.12	1.45	2.58	2.09	2.23
12	N	2	large	ProPit	Protium	pittieri	1.07	1.06	1.10	2.38	2.39	2.13
7	C	2	small	ProRav	Protium	ravenii	1.17	1.11	1.12	2.24	2.06	1.83
6	P	2	large	ProSp	Protium	Sp.	0.69	1.19	1.03	1.33	1.78	1.74
8	C	3	small	PsyPass	Psychotria	Sp.	1.11	1.15		2.70	2.64	
3	NP	1	small	RinHum	Rhinorea	hummeli	1.06	0.97	1.18	2.17	2.28	2.44

21	P	6	small	RinHum	Rhinorea	hummeli	1.40	1.41		3.03	2.87	
7	C	2	large	RyaEsp	Ryania	especiosa	1.26		1.19	2.98		2.95
8	C	3	large	RyaEsp	Ryania	especiosa	1.33	1.12	1.19	3.12	2.27	2.70
9	P	3	large	RyaEsp	Ryania	especiosa	1.00	1.03	1.05	2.62	2.74	2.47
1	N	1	small	RyaSpe	Ryania	especiosa	1.33	1.21	1.24	3.21	3.50	3.03
2	C	1	small	RyaSpe	Ryania	speciosa	1.55	0.93	1.20	1.93	2.80	2.96
3	NP	1	small	RyaSpe	Ryania	especiosa	1.14	0.78	1.15	2.97	2.37	2.47
4	P	1	small	RyaSpe	Ryania	especiosa	0.96	1.10		3.13	3.04	2.61
7	C	2	small	RyaSpe	Ryania	especiosa	1.18	0.81	1.21	2.83	2.53	2.75
8	C	3	small	RyaSpe	Ryania	especiosa	1.18	1.09	1.15	2.84		2.37
9	P	3	small	RyaSpe	Ryania	especiosa	1.12	1.20	1.14	3.23	2.60	2.78
12	N	2	small	RyaSpe	Ryania	especiosa	1.34	1.04	1.18	3.22	2.68	2.88
23	NP	6	small	Schweilera	Eschweilera	costarricensis	1.03	0.98	1.20	2.24	1.76	1.88
24	C	6	small	Schweilera	Eschweilera	costarricensis			1.04			2.19
8	C	3	small	Schweilera	Eschweilera	costarricensis			0.93			3.17
14	C	4	large	SimAma	Simarouba	amara	1.32	0.88	1.34	3.59	2.50	2.51
1	N	1	large	SocExo	Socratea	exorrhiza	1.24	1.36	1.31	2.13	2.46	2.39
1	N	1	small	SocExo	Socratea	exorrhiza	1.27	1.50	1.34	2.26	2.33	2.08
2	C	1	large	SocExo	Socratea	exorrhiza	1.21	1.32	1.30	2.20	1.67	2.67
2	C	1	small	SocExo	Socratea	exorrhiza	1.21	1.16	1.33	2.07	2.16	2.43
3	NP	1	large	SocExo	Socratea	exorrhiza	1.72	1.16	1.35	2.24	1.88	2.21
3	NP	1	small	SocExo	Socratea	exorrhiza	1.26	0.99	1.16	2.22	1.99	2.02
4	P	1	large	SocExo	Socratea	exorrhiza	1.05	1.30	1.64	2.10	1.96	2.70
4	P	1	small	SocExo	Socratea	exorrhiza	1.22	1.14	1.35	2.20	2.37	2.41
5	NP	2	large	SocExo	Socratea	exorrhiza		1.14	1.76	1.99	2.53	2.40
5	NP	2	small	SocExo	Socratea	exorrhiza	1.28	1.13	1.32	2.43	2.39	2.30
6	P	2	large	SocExo	Socratea	exorrhiza						2.07
6	P	2	large	SocExo	Socratea	exorrhiza		1.47	1.42		2.46	2.22
7	C	2	large	SocExo	Socratea	exorrhiza	1.18	1.22	1.44	1.75	1.81	1.92
7	C	2	small	SocExo	Socratea	exorrhiza		1.07	1.19			2.01
8	C	3	large	SocExo	Socratea	exorrhiza		1.53	1.46		2.34	2.41
9	P	3	large	SocExo	Socratea	exorrhiza	1.16	1.23	1.45	2.14	2.13	2.38
9	N	3	small	SocExo	Socratea	exorrhiza		2.15	1.42		2.49	2.35
10	N	3	large	SocExo	Socratea	exorrhiza	1.78	1.50	1.42	1.90	2.19	2.68

10	N	3	small	SocExo	Socratea	exorrhiza	1.38	1.36		2.11	2.31	
10	N	3	small	SocExo	Socratea	exorrhiza			1.15			2.09
11	NP	3	large	SocExo	Socratea	exorrhiza		1.52	1.47		2.38	2.66
11	NP	3	large	SocExo	Socratea	exorrhiza	1.24	1.06	1.33	2.16	2.18	2.42
11	NP	3	small	SocExo	Socratea	exorrhiza		1.35	1.29		2.51	2.26
11	NP	3	small	SocExo	Socratea	exorrhiza	1.17	1.32	1.38	1.89	2.06	2.26
12	N	2	large	SocExo	Socratea	exorrhiza	1.44	1.31		2.16	2.17	2.61
12	N	2	small	SocExo	Socratea	exorrhiza	0.80	1.39	1.23	2.19	2.03	2.18
13	N	4	large	SocExo	Socratea	exorrhiza	1.43	1.23	1.21	2.12	2.33	2.23
13	N	4	small	SocExo	Socratea	exorrhiza	1.02	1.40	1.12	2.29	2.36	1.89
14	C	4	large	SocExo	Socratea	exorrhiza	1.38	1.36	1.30	2.41	2.73	2.76
14	C	4	small	SocExo	Socratea	exorrhiza	1.14	0.93	1.29	2.27	2.07	2.32
15	P	4	large	SocExo	Socratea	exorrhiza	1.36		1.51	2.15	2.12	2.22
15	P	4	small	SocExo	Socratea	exorrhiza	1.31	1.29	1.35	2.27	2.44	2.26
16	P	5	large	SocExo	Socratea	exorrhiza	1.15	1.57	1.31	1.99	2.08	2.43
16	P	5	small	SocExo	Socratea	exorrhiza	1.58		1.49	2.12		1.99
17	N	5	large	SocExo	Socratea	exorrhiza	1.54	1.24		2.16	2.58	
17	N	5	small	SocExo	Socratea	exorrhiza	1.29	1.13		2.32	2.30	
18	NP	5	large	SocExo	Socratea	exorrhiza	1.26	1.09		2.17	2.05	3.36
18	NP	5	small	SocExo	Socratea	exorrhiza	1.33	1.29	1.27	2.39	2.22	2.27
19	C	5	large	SocExo	Socratea	exorrhiza	1.13	1.05	1.21	2.03	2.15	2.18
19	C	5	small	SocExo	Socratea	exorrhiza	0.95		1.31	2.32	2.36	2.20
20	NP	4	large	SocExo	Socratea	exorrhiza	1.03	1.17	1.22	2.20	2.52	2.36
20	NP	4	small	SocExo	Socratea	exorrhiza	0.81	1.59	1.59	2.44	2.51	2.08
20	NP	4	small	SocExo	Socratea	exorrhiza			1.45			2.12
21	P	6	large	SocExo	Socratea	exorrhiza		1.90	1.64		2.37	2.88
21	P	6	small	SocExo	Socratea	exorrhiza	1.52	1.25	1.40	2.38	2.27	2.38
22	N	6	large	SocExo	Socratea	exorrhiza	1.02	1.31		1.99	2.18	
22	N	6	large	SocExo	Socratea	exorrhiza		1.19			2.14	
22	N	6	small	SocExo	Socratea	exorrhiza	1.23	1.09		2.45	1.94	
23	NP	6	large	SocExo	Socratea	exorrhiza	1.24	1.53	1.65	2.21	2.14	2.49
23	NP	6	small	SocExo	Socratea	exorrhiza	1.53	1.25	1.27	2.35	2.49	2.04
24	C	6	large	SocExo	Socratea	exorrhiza		1.27	1.22		1.96	2.10
24	C	6	large	SocExo	Socratea	exorrhiza	1.26	1.30	1.21	2.18	2.19	2.00

24	C	6	small	SocExo	Socratea	exorrhiza		1.32	1.27		2.19	2.01
24	C	6	small	SocExo	Socratea	exorrhiza		2.00			3.05	
9	P	3	small	TalNer	Talisia	nervosa	1.28	0.92	1.22	1.78	1.63	1.83
8	C	3	large	TraAsp	Trattinnickia	aspera	1.50	1.45	1.21	2.56	2.25	2.61
13	N	4	large	TraAsp	Trattinnickia	aspera	1.28	1.31	1.37	2.35	2.34	2.37
21	P	6	large	TraAsp	Trattinnickia	aspera	1.40		1.23	2.45		2.12
22	N	6	small	TraAsp	Trattinnickia	aspera	1.51	1.97		2.72	2.98	
18	NP	5	small	VirKosch	Virola	koschynii	0.92	1.05	1.04	2.34	2.20	2.35
6	P	2	small	VirMul	Virola	multiflora	1.07	0.96	1.25	2.38	2.42	2.53
1	N	1	large	VirSeb	Virola	sebifera	1.13	1.05	1.08	2.47	2.83	2.56
8	C	3	large	VirSeb	Virola	sebifera	1.13		1.35	2.59		2.69
14	C	4	large	VirSeb	Virola	sebifera	1.40	1.19	1.14	2.70	2.90	2.67
17	N	5	large	VirSeb	Virola	sebifera	1.25	1.04		2.78	2.83	
21	P	6	large	VirSeb	Virola	sebifera	1.22	1.58	1.53	2.67	2.85	2.75
23	NP	6	small	VirSeb	Virola	sebifera	1.28	1.39	1.25	2.62	2.72	2.66
24	C	6	large	VirSeb	Virola	sebifera	1.45	1.33	0.95	2.95	2.96	2.91
24	C	6	small	VirSeb	Virola	sebifera	0.78	0.97	1.20	2.67	2.71	2.70
